# APC-Targeted DNA Vaccination Against Reticulocyte-Binding Protein Homolog 5 Induces *Plasmodium falciparum*-Specific Neutralizing Antibodies and T Cell Responses

**DOI:** 10.3389/fimmu.2021.720550

**Published:** 2021-10-18

**Authors:** Louise Bjerkan, Ganesh Ram R. Visweswaran, Arnar Gudjonsson, Geneviève M. Labbé, Doris Quinkert, David J. Pattinson, Heidi C. L. Spång, Simon J. Draper, Bjarne Bogen, Ranveig Braathen

**Affiliations:** ^1^ Institute of Clinical Medicine, University of Oslo and Oslo University Hospital, Oslo, Norway; ^2^ Jenner Institute, University of Oxford, Oxford, United Kingdom

**Keywords:** APC-targeting, DNA vaccines, PfRH5, malaria, antibody responses, T cell responses, GIA

## Abstract

Targeted delivery of antigen to antigen presenting cells (APCs) is an efficient way to induce robust antigen-specific immune responses. Here, we present a novel DNA vaccine that targets the *Plasmodium falciparum* reticulocyte-binding protein homolog 5 (PfRH5), a leading blood-stage antigen of the human malaria pathogen, to APCs. The vaccine is designed as bivalent homodimers where each chain is composed of an amino-terminal single chain fragment variable (scFv) targeting unit specific for major histocompatibility complex class II (MHCII) expressed on APCs, and a carboxyl-terminal antigenic unit genetically linked by the dimerization unit. This vaccine format, named “Vaccibody”, has previously been successfully applied for antigens from other infectious diseases including influenza and HIV, as well as for tumor antigens. Recently, the crystal structure and key functional antibody epitopes for the truncated version of PfRH5 (PfRH5ΔNL) were characterized, suggesting PfRH5ΔNL to be a promising candidate for next-generation PfRH5 vaccine design. In this study, we explored the APC-targeting strategy for a PfRH5ΔNL-containing DNA vaccine. BALB/c mice immunized with the targeted vaccine induced higher PfRH5-specific IgG1 antibody responses than those vaccinated with a non-targeted vaccine or antigen alone. The APC-targeted vaccine also efficiently induced rapid IFN-γ and IL-4 T cell responses. Furthermore, the vaccine-induced PfRH5-specific IgG showed inhibition of growth of the *P. falciparum* 3D7 clone parasite *in vitro*. Finally, sera obtained after vaccination with this targeted vaccine competed for the same epitopes as PfRH5-specific mAbs from vaccinated humans. Robust humoral responses were also induced by a similar *P. vivax* Duffy-binding protein (PvDBP)-containing targeted DNA vaccine. Our data highlight a novel targeted vaccine platform for the development of vaccines against blood-stage malaria.

## Introduction

The development of an efficient vaccine against malaria is under intense investigation and remains an important goal to control and eliminate the disease, which causes over 200 million cases leading to ~0.5 million deaths each year (1, 2). The Achilles heel of blood-stage subunit vaccine development has been considerable sequence polymorphisms in target antigens (3), and/or elicitation of antibody responses in human vaccinees of insufficient breadth for effective neutralization (4). The *Plasmodium falciparum* reticulocyte-binding protein homolog 5 (PfRH5) recently emerged as a leading candidate antigen against the blood-stage human malaria parasite (5, 6). Together with PfCyRPA and PfRipr, PfRH5 forms a stable and highly conserved complex that can induce strain-transcendent neutralizing antibodies (7). During merozoite invasion, PfRH5 binds specifically to the receptor Basigin on the human erythrocyte surface and blocking of this essential interaction can prevent invasion (8). Low natural immune pressure, coupled with functional constraints linked to Basigin binding, likely account for the limited sequence diversity of PfRH5 (9). A 45 kDa truncated version of full length PfRH5, PfRH5ΔNL, which lacks the amino-terminus (N) and internal loop (L) disordered regions was used to determine the crystal structure in complex with its host receptor Basigin (10). Similar to the full length PfRH5, this smaller version also induced growth-inhibitory immune responses, and contains the epitopes targeted by inhibitory mouse and human antibodies (10, 11). Consequently, PfRH5ΔNL has become a promising candidate for structure-based vaccine design (12). The goal of blood-stage vaccines is to induce antibodies against the merozoite form of the parasite that invades erythrocytes, and restrict parasite replication in the blood, protect against disease severity and/or reduce transmission by accelerating the control and clearance of blood-stage parasitemia (4). Very rapid erythrocyte invasion and thus a limited exposure window of merozoite antigens to antibodies imposes kinetic constraints on neutralization of the parasite. Consequently, a high concentration of functional antibody appears necessary to achieve neutralization, and the need of efficient vaccines that induce high amounts of antigen-specific antibodies remains urgent (6, 13, 14).

Traditional approaches to antibody induction by vaccination have included the delivery of recombinant protein and particle-in-adjuvant formulations, and more recently, the use of recombinant viral vectored vaccines to deliver protein antigens of interest (6, 15, 16). DNA vaccines have attractive properties due to their highly versatile format, ease of construction, robust and rapid production, low-cost manufacturing, and *in situ* antigen production (17). However, a drawback with naked DNA vaccines is poor immunogenicity of encoded antigens (18, 19). Targeting of antigens to surface molecules on antigen presenting cells (APCs) is a strategy to substantially increase immune responses after DNA immunization (20). APC targeting by the Vaccibody technology has been explored in mice for various diseases like multiple myeloma (21) and HIV (22). Targeting of antigen to major histocompatibility complex class II (MHCII) molecules expressed on APCs are shown to be especially efficient for induction of protective immune responses against influenza in mice (23–25), but also in larger animals like ferrets, pigs (26) and rhesus macaques (27). The MHCII-targeting leads to an increased germinal center reaction with more MHCII-loaded peptides, germinal center B cells, T_FH_ cells and plasma cells (28). To the best of our knowledge, delivery of MHCII-targeted DNA vaccines against malaria antigens has not been explored to date.

Here, we examine antibody and T cell responses induced by this vaccination strategy, where DNA plasmids were used that encode homodimeric proteins targeting the PfRH5ΔNL antigen to MHCII molecules expressed on APCs. As non-targeted controls, we included plasmids encoding antigen alone or linked to a scFv specific for the hapten NIP. We tested the functional ability of PfRH5-specific serum antibodies from mice to inhibit 3D7 clone *Plasmodium falciparum* parasites *in vitro* and looked for reactivity towards previously defined epitopes critical for human antibody responses against PfRH5. Finally, to demonstrate the versatility of this format, we also introduced a second malaria antigen, the *Plasmodium vivax* Duffy-binding protein (PvDBP), into this format and assessed its ability to elicit PvDBP-specific antibody responses.

## Materials and Methods

### Animals and Cell Lines

Female BALB/c mice (Janvier Labs, Le Genest-Saint-Isle, France) 6-8 weeks old were used for the experiments. The mice were housed under minimal disease conditions at Oslo University Hospital (Oslo, Norway). All experiments were reviewed and approved by the Norwegian Animal Research Authority and were carried out in accordance with the recommendations from the Guide for the Care and Use of Laboratory Animals of the Norwegian National Institute of Health. Cell work was performed with human embryonic kidney (HEK) 293E cells purchased from the American Type Culture Collection (Manassas, VA, USA). Cell lines were cultured in complete cell culture medium RPMI 1640 (Invitrogen) containing 10% heat inactivated FCS (Life Technologies), 24 mg/L gentamicin (Sanofi-Aventis Norge AS), 50 µM monothioglycerol (Sigma), 1 mM sodium pyruvate (Lonza), and 0.1 mM non-essential amino acids (Lonza).

### Construction of DNA Vaccine Molecules

DNA vaccines were encoded in a pUMVC4a plasmid vector (Aldevron) under a CMV-IE promoter and containing a tPA signal peptide. The tPA (A22P) (29), was amplified with PCR using the primers: F-tPA: 5`ctgggtacccacgtgACCTCACCATGGATGCAATGAAGAGAGGGCTCTGCTGTGTGCTGCTG-3`, R-tPA: 5`gtcg*gaatgca*cGCTTGCCGAAACGAAGACTGCTCCACACAGCAGCAGCACACAGCAGAGCC-3` (KpnI, PmlI and *BsmI* restriction enzyme sites are included, the tPA leader sequence in capital letters) and cloned into Vaccibodies in the pLNOH2 plasmid vector (20) using KpnI and BsmI restriction enzyme sites. The tPA (A22P, amino acids MDAMKRGLCCVLLLCGAVFVSAS) replaced the signal peptide sequence from the V_H_ gene of B1-8 mAb (30). The targeted constructs were cloned into pUMVC4a using PmlI and BamHI. For targeting units single chain fragment variable (scFv) specific for MHCII (scFv^αI-Ed^, from mouse 14-4-4S anti-MHCII mAb specific for the Ia.7 determinant on I-Ed molecules), or for the hapten 4-hydroxy-3-iodo-5-nitrophenylacetic acid (scFv^αNIP^, non-targeted control) were used. The dimerization unit was composed of a human Ig-hinge regions h1 and h4 and C_H_3 domain from human γ3 chains (20). The antigens PfRH5ΔNL or region II of the PvDBP (hereafter referred to as “PvDBP”) where amplified with specific primers; F-PfRH5ΔNL: 5`AAAGGCCTCAGCGGCCTGGGTACCAAGAACGTGAACTTCCTG-3`, R-PfRH5ΔNL: 5`CCGGCCCTGCAGGCCTCATCAGGATCCCTGGGTCAGGGGTT-3`, F-PvDBP: 5`AAAGGCCTCAGCGGCCTGGACCACAAGAAAACCATCAG-3`, R-PvDBP: 5`CCGGCCCTGCAGGCCTCATCAGGTGACGACTTCCTGGGTGTT-3` (the SfiI sites underlined) and subcloned into the conventional Vaccibody using the SfiI-SfiI sites. The PfRH5ΔNL or PvDBP antigen alone were amplified with the primers: F-PfRH5: 5`ttcacgtgACCTCACCATGGACGCTATGAAGAGGGGCCTGTGC-3` (PmlI site underlined), and R-PfRH5ΔNL and R-PvDBP primer above with SfiI site included and cloned into pUMVC4a using PmlI-SfiI restriction enzyme sites, leaving the antigens with a the tPA signal peptide (amino acids MDAMKRGLCCVLLLCGAVFVSPSQEIHARFRR). For the construction of reversed Vaccibody constructs, PfRH5ΔNL was amplified with primers; F-PfRH5ΔNL: 5`ttcacgtgACCTCACCATGGACGCTATGAAGAGGGGCCTGTGC-3`(PmlI site underlined), R-PfRH5ΔNL: 5`gctag*cgtacg*GGATCCCTGGGTCAGGGGTTTGTTC-3` (*BsiWI* included), and PmlI and *BsiWI* restriction enzymes were used to replace the signal peptide and targeting units with the antigen containing the signal peptide similar to antigen alone. Similarly the scFv^αI-Ed^ and scFv^αNIP^ were amplified with the following primers: F-scFv^αNIP^: 5`AAAGGCCTCAGCGGCCTGCAGGCTGTTGTGACTCAGGAATCTGC-3`, R-scFv^αNIP^: 5`CCCCGGCCCTGCAGGCCTCATCATGAGGAGACTGTGAGAGTGGTGC-3`, F-scFv^αMHCII^: 5`AAAGGCCTCAGCGGCCTGCAGGTCCAATTGACACAGTCTCCTGC-3`, R-scFv^αMHCII^: 5`CCCCGGCCCTGCAGGCCTCATCATGAGGAGACGGTGACTGAGGTTCC-3` (SfiI sites underlined), and *via* SfiI-SfiI cutting replacing the antigen.

### ELISA for *In Vitro* Assessment of Vaccine Proteins

1x10^5^ HEK293E cells per well were seeded in 24 well culture plates (Corning), and incubated at 37°C with 5% CO_2_. The following day, the cells were transiently transfected with 1.0 µg DNA plasmid with 2 µg Polyethylenimine (Polysciences). Supernatants were collected after 3-4 days, and centrifuged 5 min at 2000 rpm. High-binding 96-well plates (Costar 3590, Corning) were coated in PBS with either the anti-PfRH5 mouse mAb (9AD4, 0.5 µg/mL) (31) or mouse anti-human C_H_3 (1:1000, MCA878G, Sigma), incubated at 4°C overnight and blocked with blocking buffer (PBS with 1% w/v BSA and 0.02% w/v Na-Azide) per well. Undiluted supernatants from HEK293E cells were added in triplicates, and vaccine proteins were detected with either (1) rabbit anti-PfRH5FL (full-length) sera (rabbit αRH5 pAb, 1:1000) (31); (2) one of the mouse anti-PfRH5 mAbs QA1, QA5, 2AC7, 8BB10, 9AD4 or 4BA7 (1 µg/mL, 0.5 µg/mL, 1 µg/mL, 0.24 µg/mL, 0.5 µg/mL and 3 µg/mL, respectively) (31); or (3) rabbit anti-PvDBP sera (1:1000) (32). Rabbit pAb sera (anti-PfRH5 or anti-PvDBP) were detected with goat anti-rabbit IgG Alkaline Phosphatase (AP)-conjugated Ab (1:2000, Sigma-Aldrich). Mouse anti-PfRH5 mAbs were detected by biotin-conjugated anti-IgG1 (QA5, 2AC7 and 8BB10) or anti-IgG2a (QA1, 4BA7 or 9AD4) followed by streptavidin-AP (1:5000) (GE Healthcare). The ELISAs were developed with phosphatase substrate (Sigma-Aldrich) dissolved in diethanolamine substrate buffer and OD_405nm_ was measured using a TECAN microplate reader.

### Vaccination

Mice were anesthetized by intraperitoneal (i.p.) injection with 0.1 mg/10 g body weight with a cocktail of; Zoletil Forte (250 mg/ml, Virbac), Rompun (20 mg/ml, Bayer Animal Health GMBH), and Fentanyl (50 µg/ml, Actavis). All DNA vaccines were purified by EndoFree Plasmid Mega Kit (Qiagen) and dissolved in sterile 0.9% NaCl solution (B. Braun). For intramuscular (i.m.) delivery of DNA vaccines, mice were shaved on each leg, and 25 µg of PfRH5ΔNL-containing DNA or 2.5 µg of PvDBP-containing DNA was injected in a 50 µl volume into each quadriceps femoris muscle (50 µg or 5 µg total DNA/mouse, respectively). Immediately after injection, electrical pulses were applied at the injection site (Elgen 1000 Needle Electroporator, Inovio Biomedical Co., Blue Bell, PA, USA) as described (24). 1x10^10^ infectious units (IU)/mouse were injected i.m. with AdHu5 containing PfRH5FL (33) in the adenoviral boost.

### Serum ELISAs

Blood samples were collected from the saphenous vein, and sera isolated by two successive centrifugations for 5 min at 13.000 rpm. The serum ELISAs were run as described above for vaccine protein ELISAs except for the following. Plates were coated with PfRH5FL protein (also known as RH5.1) (34) or PvDBP protein (6) (1 µg/ml in PBS, 0.02% w/v Na-azide) and sera were serially diluted 3-fold in ELISA buffer (PBS with 0.2% Tween20, 0.1% BSA, and 0.02% Na-azide) starting at 1:50. Polyclonal Abs in mouse sera were detected with either goat anti-mouse IgG-AP conjugated Ab (1:5000, A2429; Sigma-Aldrich), biotinylated mouse anti-mouse IgG1^[a]^ (1 µg/ml, 553500; BD Pharmingen), or biotinylated mouse anti-mouse IgG2a^[a]^ (1 µg/ml, 553502; BD Pharmingen) followed by streptavidin-AP (GE Healthcare). The Ab titer was defined as the last serum dilution giving an absorbance above background (mean + 3×SD) of NaCl-vaccinated mice. Samples with a titer <50 were given an endpoint titer of 10 designated as n.d. (not detected).

### Inhibition ELISA

Sera (diluted 1:150) were incubated with various combinations of human anti-PfRH5 mAbs (R5.011, R5.016, R5.008, R5.001, R5.015, and/or R5.004) (11), at concentrations ranging from 0.08 µg/ml - 50 µg/ml per competing mAb in binding buffer (PBS with 1% BSA and 0.2% Tween20) for 1 hour at room temperature. R5.007 (300 µg/ml), which do not binds PfRH5ΔNL (11), or a human mAb IgG1 isotype control (0.08 µg/ml - 50 µg/ml, BioCell) were added as controls. Serum-mAb mixes were added in triplicate to plates coated with PfRH5FL protein (1 µg/ml in PBS, 0.02% w/v Na Azide) and blocked with 0.1% BSA in PBS. Plates were incubated overnight at 4°C with the mixtures, and PfRH5FL-specific mouse IgG was subsequently detected with goat anti-mouse IgG-AP as described above. OD_405nm_ signal was measured and the relative reduction (%) of OD_405nm_ signal compared to sera that was pre-incubated with binding buffer only (without mAbs) was calculated.

### ELISPOT Assay

Mouse IFN-γ or IL-4 ELISpot PLUS plates (Mabtech, Nacka Strand, Sweden) were used according to the manufacturer’s protocol for analyzing IFN-γ or IL-4 secreting T cells. In short, mice spleens and draining lymph nodes (dLNs, lumbar and sacral) were harvested and single cell suspensions were prepared with the gentleMACS™ Dissociator (Milteny Biotech, Bergisch Gladbach, Germany). Single cells were incubated in Tris-buffered ammonium chloride (ACT) erythrocyte lysis buffer for 5 min on ice followed by filtration through a 75 µm Nylon strainer. Splenocytes were seeded at a concentration of 2.5 × 10^5^ cells per well and restimulated with PfRH5FL protein (34); or specific individual peptides previously identified to contain H-2^d^ T cell epitopes within PfRH5: F10 (NDVPIKMEYFQTYKKNKPLT), G10 (DVPIKMEYFQTYKKNKPLTQ), F6 (KHLSYNSIYHKSSTYGKCIA) and D9 [YIDTIKFIHKEMKHIFNRIE, (34)], or irrelevant peptide (IYSTVASSL, MHC class I restricted from hemagglutinin) at 5 µg/ml. Plates were incubated for 18 h at 37°C in 5% CO_2_, before incubation with detection antibody (IFN-γ detection: 3321-4APT or IL-4 detection: 3311-4APW, Mabtech) and developed until distinct spots appeared with the BCIP^®^/NBT-Purple Liquid Substrate System for Membranes (B3679, Sigma-Aldrich). Spots were automatically counted and analyzed with a CTL ELISPOT reader (CTL Europe, Bonn, Germany). Number of spots in wells stimulated with irrelevant peptide were subtracted from the number of spots in PfRH5-stimulated wells.

### Assay of Growth Inhibitory Activity (GIA)

The assays of GIA were performed at the Jenner Institute, University of Oxford using previously described methodology (34). In brief, total IgG was purified using protein G and incubated with synchronized *P. falciparum* late trophozoites in culture. Relative parasitemia levels were quantified by colorimetric determination of parasite lactate dehydrogenase after one complete life-cycle (40-44h).

### Statistical Analysis

Data were analyzed using GraphPad Prism 8 (GraphPad Software, San Diego, CA, United States). Two-tailed Mann-Whitney test was performed to compare mean antibody and T cell responses between groups. A value of p < 0.05 was considered significant.

## Results

### Construction of APC-Targeted DNA Vaccines Containing PfRH5 Antigen

**Figure 1:**
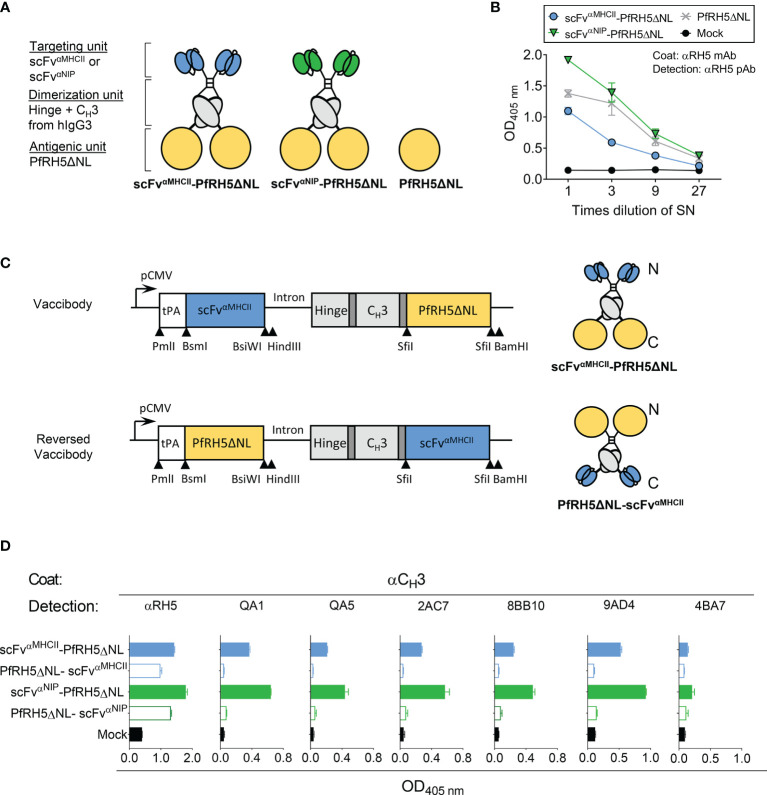
Characterization of APC-targeted malarial DNA vaccines and translation into vaccine proteins. **(A)** Schematic representation of dimeric Vaccibody proteins. Each chain of the dimer was composed of a targeting unit consisting of a scFv that binds MHC class II (scFv^αMHCII^, blue) molecules on APCs or a non-targeted control (scFv^αNIP^, green), a dimerization unit (grey) composed of a shortened Ig hinge and CH3 domain from human γ3 chains, and the truncated PfRH5ΔNL as the antigenic unit (yellow). In addition, a vaccine plasmid only expressing the PfRH5ΔNL was included. **(B)** HEK293E cells were transiently transfected with DNA plasmids as indicated, and vaccine proteins containing PfRH5ΔNL in the supernatants (SN) were detected by sandwich ELISA. **(C)** In the conventional Vaccibody (upper right), the antigenic unit was coupled at the C-terminal end of the fusion protein. Whereas for the reversed Vaccibody (lower right), the antigenic unit was linked N-terminally to the dimerization unit. The DNA vaccine plasmids (left, upper and lower construct) contain a promoter (pCMV), a tPA leader peptide (white box) and short linkers (dark grey) that increase the flexibility of the various units. Unique restriction enzyme sites for cloning are indicated. **(D)** PfRH5ΔNL expressed as targeted or non-targeted vaccines either as a C-terminal fusion (conventional Vaccibody) or N-terminal fusion (reversed Vaccibody) in HEK293E cells, and detected with αPfRH5 pAb or various unique anti-PfRH5 mAbs. Data shown are mean ± SD.

The APC-targeted vaccine construct, termed a “Vaccibody”, was designed as a bivalent homodimeric molecule. Each chain contains an amino-terminal (N-terminal) targeting unit, a human IgG3-derived dimerization unit and a carboxyl-terminal (C-terminal) antigenic unit (**Figures 1A, C**, upper constructs). The shortened hinge and Cγ3 domain from human IgG3 cause dimerization, thereby producing covalent dimeric molecules *via* disulfide bonds within the hinge. The N-terminal scFv is specific for and binds mouse MHCII (scFv^αMCHII^) molecules on APCs. A non-targeted control vaccine was also prepared with a scFv specific for the hapten NIP (scFv^αNIP^) as the targeting unit; the hapten NIP should not be present in the body. The shorter form of PfRH5 that encompasses amino acid residues 140-526 but lacks 248-296 (PfRH5ΔNL), shown to retain binding to the receptor Basigin (8, 10), was cloned into the antigenic unit, giving rise to our targeted vaccine designated scFv^αMHCII^-PfRH5ΔNL. Finally, a control plasmid only expressing PfRH5ΔNL antigen was also constructed (**Figure 1A**).

To evaluate the efficiency of translation into Vaccibody proteins, vaccine-encoding plasmids were transiently transfected into HEK293E cells, and vaccine proteins in supernatants were tested in ELISA with conformation-dependent mAb. DNA vaccines containing PfRH5ΔNL (**Figure 1B**) were efficiently translated into vaccine proteins and recognized by capture with a PfRH5-specific mAb (9AD4) and detection with anti-PfRH5 pAb rabbit serum. The binding of PfRH5-specific Abs suggested the conformation of PfRH5ΔNL to be retained when expressed as a fusion protein within the Vaccibody. To further evaluate the structure of PfRH5ΔNL as a fusion protein, a panel of mouse mAbs (QA1, QA5, 2AC7, 8BB10, 9AD4 and 4BA7), previously characterized for PfRH5 binding and parasite growth-inhibitory activity (31), were used. All mAbs showed reactivity against PfRH5ΔNL-containing vaccine protein, except 4BA7 which is specific for PfRH5FL only, i.e. whose epitope is absent in PfRH5ΔNL (**Figure 1D**). This indicated intact epitope integrity of PfRH5ΔNL antigen when associated with the Vaccibody molecule. In conclusion, the PfRH5ΔNL was correctly folded and retained known B cell epitopes as a fusion Vaccibody protein, and could be expressed as an APC-targeted DNA vaccine.

### N-Terminal Linking of Malaria Antigen to the APC-Targeted DNA Vaccines Is Essential for Optimal Expression and Folding

Due to the buried location of the N-terminus in PfRH5ΔNL (10), N-terminal coupling of PfRH5ΔNL could potentially obscure PfRH5 inhibitory epitopes when the antigen is genetically fused to the dimerization unit of the Vaccibody. We therefore fused the PfRH5ΔNL C-terminally and the targeting units N-terminally to the dimerization unit, resulting in a “reversed Vaccibody” molecule (**Figure 1C**, lower construct). These constructs were transiently transfected into HEK293E cells and the expression of the reversed vaccine molecules in supernatant were evaluated by ELISA (**Figure 1D**). Detection by the αRH5 pAb sera demonstrated some expression of the reversed vaccines, although the expression level was reduced as compared to the conventional Vaccibodies. While all anti-PfRH5 mAbs, with the exception of 4BA7, recognized the PfRH5ΔNL-containing conventional Vaccibodies, they failed to recognize the equivalent reversed vaccines. Similar results were shown for MHCII-targeted and non-targeted vaccines (**Figure 1D**). These results suggested that the N-terminal linking of PfRH5ΔNL did not block the availability of these known B cell epitopes. Thus, N-terminal linking of this antigen is preferred as opposed to C-terminal linking.

### Targeted PfRH5-Containing DNA Vaccination Increase PfRH5FL-Specific IgG1 Antibody Responses

**Figure 2:**
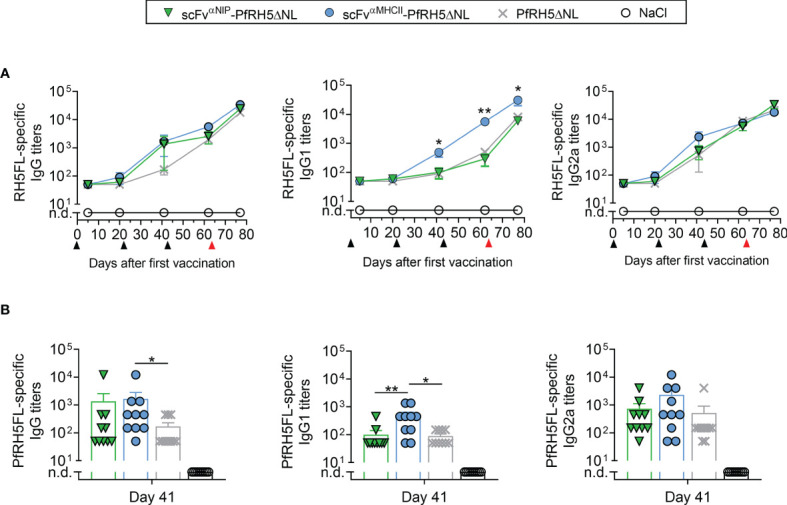
scFv^αMHCII^-PfRH5ΔNL DNA vaccination increased RH5FL-specific IgG1 antibody responses. **(A, B)** BALB/c mice were vaccinated three times (day 0, 21 and 42, black arrows) with 50 µg DNA i.m./EP with scFv^αMHCII^-targeted or non-targeted (scFv^αNIP^) vaccine plasmids containing PfRH5ΔNL, the PfRH5ΔNL antigen alone or NaCl as indicated. At day 63, the mice were boosted with a PfRH5FL-adenoviral vector (i.m., 1x1010 IU/mouse, red arrow) **(A)** Serum samples were assayed for PfRH5FL-specific IgG, IgG1 and IgG2a at day 5, 20, 41, 62 and 77 in ELISA, shown as mean ± SEM. Stars indicate statistical significance between the scFv^αMHCII^-PfRH5ΔNL group and the PfRH5ΔNL antigen alone group. **(B)** Individual mice and mean ± SEM at day 41 are shown, n = 10/group (DNA vaccinated) or n = 5/group (AdHu5-PfRh5FL boosted), *p < 0.05, **p < 0.01, two-tailed Mann-Whitney test. n.d., not detectable.

BALB/c mice were immunized three times at three weeks intervals, with 50 µg plasmids that encoded either MHCII-targeted or non-targeted Vaccibodies, or antigen alone. Plasmids were administered i.m. followed by electroporation (EP) to increase the uptake of DNA by cells in the injected tissue. Serum IgG, IgG1 and IgG2a antibody titers against PfRH5FL were followed for 9 weeks by ELISA. Vaccination with the MHCII-targeted vaccine showed significantly increased levels of total PfRH5FL-specific IgG compared to vaccination with the antigen alone at days 41 and 62 post-prime vaccination (**Figure 2A**). Similarly, by assessing IgG subclass-specific responses, significantly higher levels of PfRH5FL-specific IgG1 responses were induced by the targeted vaccine as compared to antigen alone, whereas comparable levels of PfRH5FL-specific IgG2a were detected (Figure 2A). Furthermore, a tendency of higher PfRH5FL-specific IgG and IgG2a titers in MHCII-targeted vaccinated mice compared to non-targeted vaccinated mice, whereas a significantly higher PfRH5FL-specific IgG1 response was found at day 41 (Figure 2B), demonstrating a clear targeting effect. Thus, MHCII-targeted vaccination with PfRH5ΔNL skewed the response towards the Th2-associated IgG1 subclass and showed accelerated induction of higher responses as compared to both the non-targeted and the PfRH5ΔNL antigen alone vaccine.

To explore a DNA-viral vectored boost vaccination regimen, one group of mice from each vaccination group receiving the various DNA plasmids, including the NaCl control group, was boosted with an adenovirus of the human serotype 5 (AdHu5) encoding the full length of PfRH5 (PfRH5FL) at day 63. At day 77, the levels of IgG1 elicited by MHCII-targeted vaccine were further increased and stronger compared to the levels induced by the non-targeted and PfRH5ΔNL vaccines (**Figure 2A**), while the level of IgG2a and total IgG was comparable to that induced by control vaccines (**Figure 2A**). Thus, a heterologous booster vaccination with AdHu5-PfRH5FL induced strong PfRH5FL-specific IgG1 responses that were increased above the levels induced by non-targeted control vaccines.

### Vaccine-Induced PfRH5-Specific Serum Antibodies Compete for Epitopes Defined by Functional Growth Inhibitory Anti-PfRH5 Human Antibodies

**Figure 3:**
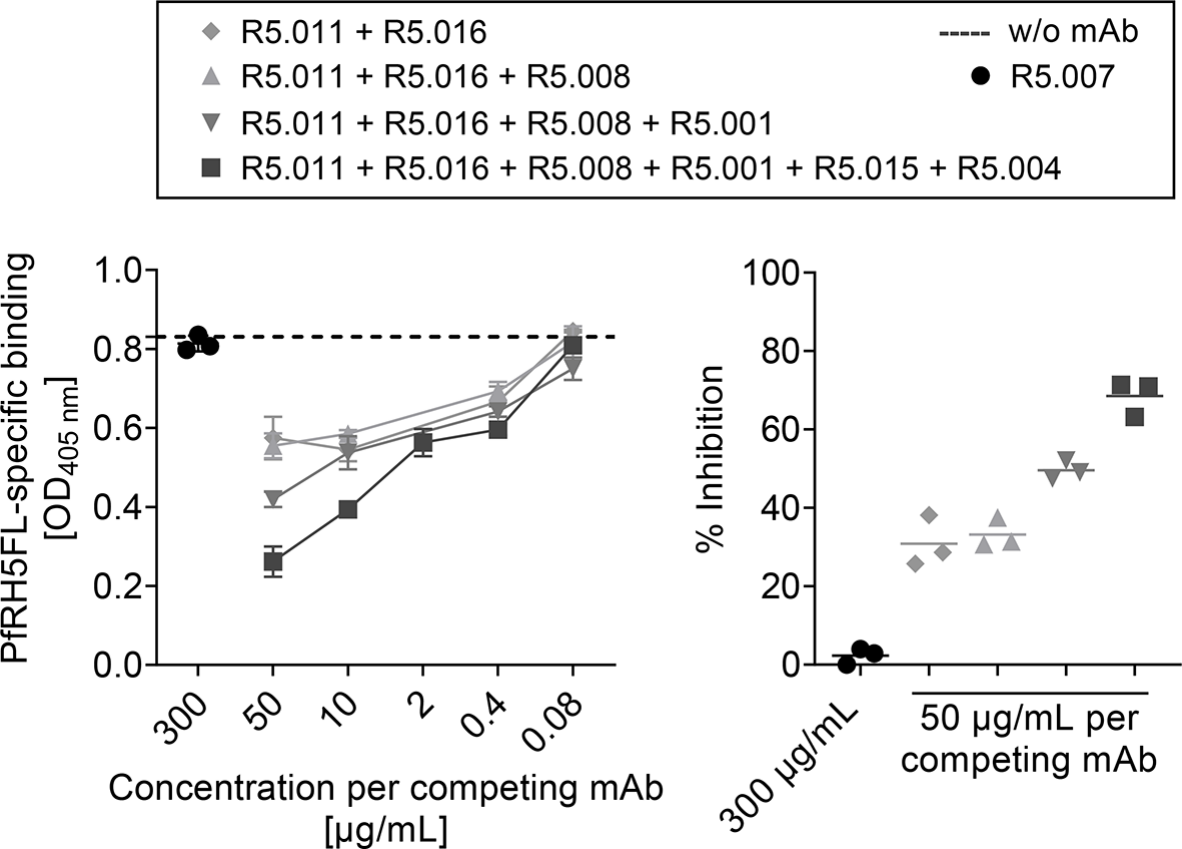
PfRH5FL-specific antibodies raised by vaccination with scFv^αMHCII^-PfRH5ΔNL DNA vaccine competed for epitopes recognized by PfRH5-specific human mAbs. BALB/c mice (n = 6) were vaccinated three times (day 0, 21 and 42) with 50 µg DNA i.m./EP with scFv^αMHCII^-PfRH5ΔNL and sera harvested at day 62. Pooled sera were incubated with a titration (50, 10, 2, 0.4 and 0.08 µg/ml per mAb) of competitor human anti-PfRH5ΔNL mAbs as indicated. Binding of total IgG in sera to PfRH5FL was measured (left panel). PfRH5FL-specific total IgG in serum without human mAbs added was indicated (stippled line). Percent (%) inhibition of binding of total IgG in sera to PfRH5FL by competing mAbs (50 µg/mL per mAb) compared to non-competing mAb (R5.007, 300 µg/mL) was calculated (right panel). All samples were performed as technical triplicates (mean ± SD).

A panel of human PfRH5-specific mAbs was obtained from the first clinical trial with a PfRH5-based vaccine (6, 11). Among these are mAbs shown to: (1) be highly growth inhibitory against 3D7 clone *P. falciparum* parasites *in vitro* in the assay of GIA (R5.016, R5.004, R5.008), some by directly blocking Basigin binding, e.g. R5.004, or others by binding in the proximity of the Basigin binding site, e.g. R5.016; or (2) to be non-inhibitory (GIA-low) but able to potentiate other growth inhibitory antibodies through a synergistic effect, e.g. R5.011, or simply non-inhibitory, e.g. those that block *in vitro* binding of PfRH5 to one of its co-proteins, PfCyRPA (R5.007, R5.001 and R5.015) (11). To test whether the PfRH5-specific mouse polyclonal antibodies generated following vaccination with our targeted vaccine bound the epitopes defined by the human mAbs, a competitive inhibition assay was conducted. The human mAbs were tested against PfRH5-specific serum raised following vaccination three times with MHCII-targeted vaccines and harvested from mice three weeks after the second boost. Combinations of human mAbs specific for epitopes located at separate sites in PfRH5ΔNL were tested to determine whether they showed competitive inhibition in an additive manner. The combination of two mAbs, R5.011 and R5.016, was sufficient to observe competitive inhibition (30.8%), while the addition of a third mAb (R5.008) had no further significant effect. Some improvement of inhibition was detected by the addition of a fourth mAb (R5.001) at the highest concentration tested (49.6%), however a mix of six human mAbs showed the strongest effect (68.5%) (Figure 3, right panel). This suggested the PfRH5ΔNL response to be specific against a broad range of epitopes on PfRH5.

As a control, we also tested the competitive binding of mAb R5.007 whose epitope was previously mapped to the internal disordered loop that is removed in PfRH5ΔNL (11). As expected, R5.007 did not inhibit the binding of vaccine-induced PfRH5-specific sera to the PfRH5FL coat at the highest concentration (**Figure 3**). An additional human IgG1 isotype control was also included at every concentration tested and showed no significant effect (**Figure S1**). Collectively, these observations suggest that APC-targeted vaccination induces a broad polyclonal serum IgG antibody response against PfRH5, including clones that are of functional relevance and have features of a desirable human antibody response against PfRH5.

### MHCII-Targeted DNA Vaccination Induces Rapid PfRH5-Specific T Cell Responses

**Figure 4:**
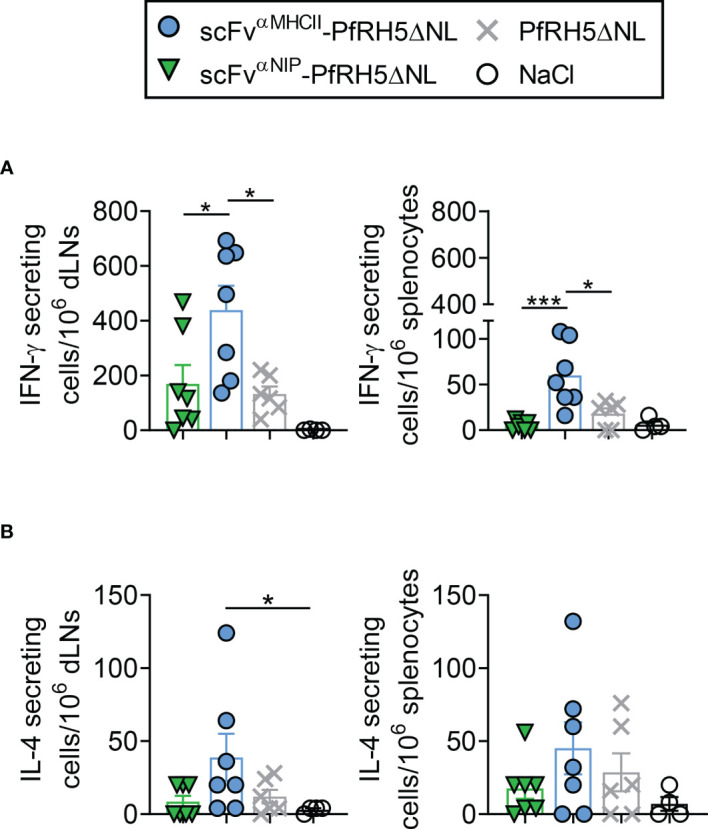
scFv^αMHCII^-PfRH5ΔNL DNA vaccination induced rapid IFN-γ and IL-4 T cell responses. **(A, B)** BALB/c mice were vaccinated once with 50 µg i.m./EP as indicated or NaCl control. Draining lymph nodes (dLN, left) or spleens (right) were harvested at day 5 post-vaccination. IFN-γ secreting cells **(A)** or IL-4 secreting cells **(B)** after stimulation with PfRH5FL protein for 48 h were analyzed (mean ± SEM). n = 7 for scFv^αNIP^-PfRH5ΔNL and scFv^αMHCII^-PfRH5ΔNL, n = 6 for PfRH5ΔNL, and n = 4 for NaCl group, *p < 0.05, ***p < 0.0001, two-tailed Mann-Whitney test.

To address antigen-specific T cell responses induced by vaccination, we analyzed the magnitude of the PfRH5-specific T cell response following vaccination with targeted and non-targeted Vaccibody DNA plasmids. Mice were vaccinated once i.m. with 50 µg PfRH5ΔNL-containing DNA vaccines followed by EP. T cell responses were assessed at day 5 post-vaccination by *ex-vivo* IFN-γ and IL-4 ELISpot assay. Splenocytes and draining lymph nodes (dLNs) were harvested and restimulated *in vitro* with PfRH5FL protein. The results showed that vaccination with the MHCII-targeted-PfRH5ΔNL vaccine induced significantly higher levels of IFN-γ producing cells in response to PfRH5FL protein in both spleens and dLNs as compared to vaccination with the non-targeted control vaccine and PfRH5ΔNL antigen alone (**Figure 4A**). Increased levels of IFN-γ producing splenocytes following vaccination with the MHCII-targeted-PfRH5ΔNL vaccine were also detected in response to the individual PfRH5 20mer peptides previously identified to contain H-2^d^ T cell epitopes (F10, G10, F6, and D9) (**Figure S2**). We also detected MHCII-targeted vaccine-induced PfRH5-specific IL-4 T cell responses in dLNs at day 5 post-vaccination, suggesting the involvement of vaccine-induced activation of Th2 cells (**Figure 4B**, left panel). Neither the non-targeted control vaccine, nor the antigen alone vaccine induced IL-4 responses above background levels at this time point in dLNs. A tendency of increased PfRH5-specific IL-4 responses was also detected in spleens (**Figure 4B**, right panel). Thus, PfRH5-specific T cell activation by the targeted vaccine is rapid and more efficient at an early time point compared to non-targeted DNA vaccines.

### Vaccine-Induced Antibodies Inhibit the Growth of *P. falciparum* 3D7 Clone *In Vitro*

**Figure 5:**
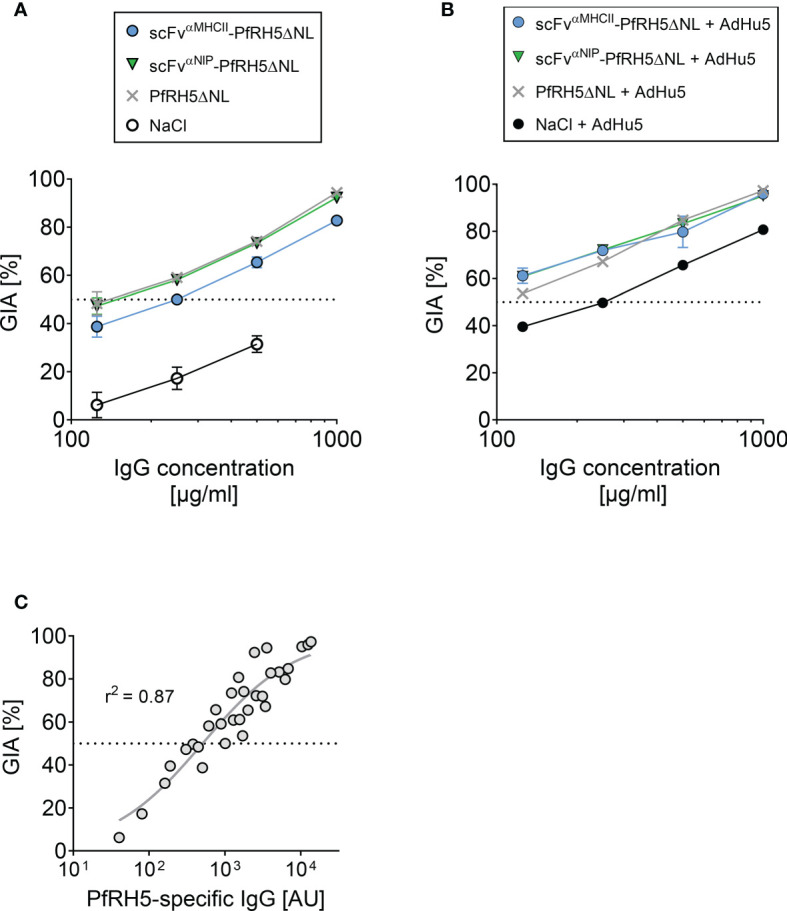
Vaccine-induced antibodies showed in vitro growth inhibition activity against 3D7 clone P. falciparum parasites. **(A, B)** In vitro growth inhibition activity (GIA) of purified IgG was assessed against 3D7 clone P. falciparum parasites. Dilution series of purified IgG from mice vaccinated i.m./EP three times (at day 0, 21, and 42) with indicated DNA plasmids or NaCl **(A)**, and from mice with an additional PfRH5FL-adenoviral boost at day 63 (i.m., 1x1010 IU/mouse) **(B)**. Sera were harvested at day 77 post-prime vaccination. The GIA assay was performed on pooled sera from n = 5 mice/group. **(C)** Relationship between GIA (%) for the dilutions shown in (A) and PfRH5FL-specific IgG titers as measured by ELISA in the purified IgG test samples. 50% GIA is marked (stippled line). Non-linear regression curve is shown (grey line, r2 = 0.87, n = 32).

We subsequently assessed the functional ability of vaccine-induced antibodies to neutralize parasites by using the standardized *in vitro* growth inhibition activity (GIA) assay. Sera were collected from mice (vaccinated 3 times at 21 day intervals) at 5 weeks after final vaccination (**Figure 5A**). Total IgG was purified from each sample and tested at various concentrations against the 3D7 clone parasites. We confirmed that GIA decreased as purified IgG was diluted in the assay. Strong and comparable levels of GIA were detected for IgG raised following DNA vaccination with PfRH5ΔNL-containing vaccines and the PfRH5ΔNL control vaccine. Similar results were obtained for total IgG purified from mice vaccinated 3 times with DNA and followed by a viral vector AdHu5-PfRH5FL boost at 21 day intervals, and serum harvested at day 77 after first vaccination (**Figure 5B**). Overall, increased levels of GIA was detected in all groups in response to the viral vector boost as compared to the three times DNA vaccination regimen alone, and all groups reached comparable GIA. In agreement with previous studies (6, 13, 35, 36), the GIA was related to PfRH5-specific IgG levels in the purified IgG test samples (**Figure 5C**). Therefore, PfRH5-specific IgG raised following APC-targeted vaccination are functional against 3D7 clone parasites.

### APC-Targeting DNA Vaccines Containing PvDBP Induce Antigen-Specific Antibody Responses

**Figure 6:**
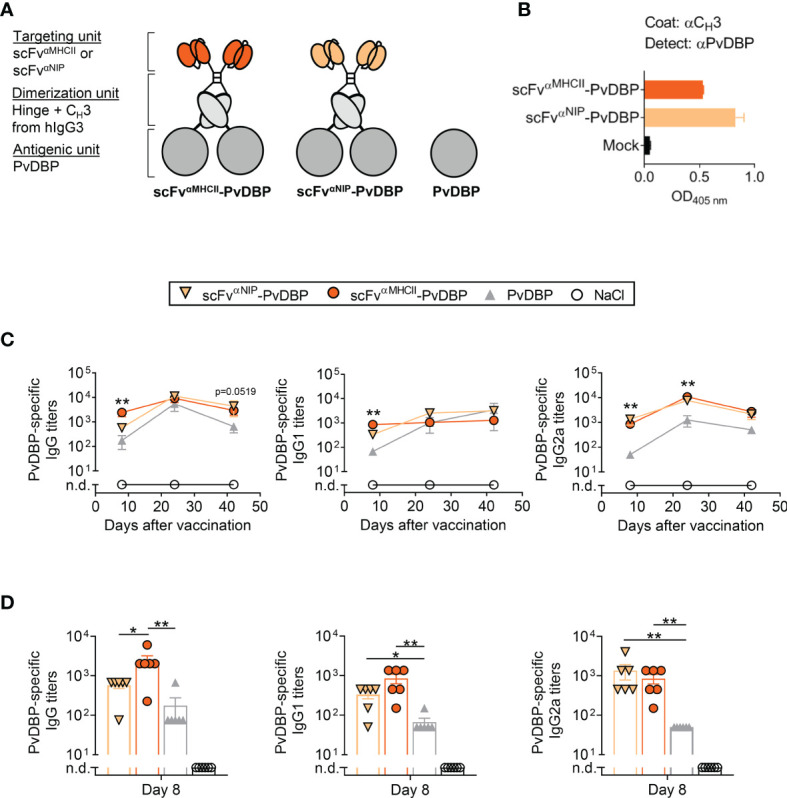
PvDBP-containing scFv^αMHCII^-targeted DNA vaccines induced antigen-specific antibody responses in mice after one vaccination. **(A)** Schematic representation of dimeric PvDBP-containing vaccine proteins. The targeted vaccines (scFv^αMHCII^, targeting unit orange), the non-targeted control (scFv^αNIP^, yellow), and the PvDBP as the antigenic unit (dark grey). In addition, a vaccine plasmid only expressing the PvDBP was included. **(B)** HEK293E cells were transiently transfected with scFv^αMHCII^–targeted and non-targeted (scFv^αNIP^) DNA plasmids containing PvDBP, and vaccine proteins in supernatant were detected by indicated sandwich ELISAs. **(C, D)** BALB/c mice were vaccinated once with 5 µg DNA i.m./EP with the indicated DNA vaccines or NaCl. **(C)** Serum samples were assayed for PvDBP-specific total IgG, IgG1 and IgG2a at day 8, 24, and 42 against PvDBP in ELISA, shown as mean ± SEM. Stars indicate statistical significance between the scFv^αMHCII^-PvDBP group and the PvDBP antigen alone group. **(D)** Individual mice and mean ± SEM at day 8 are shown, n = 6/group, *p < 0.05, **p < 0.01, two-tailed Mann-Whitney. n.d., not detectable.

To demonstrate the versatility of the APC-targeted DNA vaccine format, we also tested another malaria antigen, PvDBP, as MHCII-targeted and non-targeted Vaccibodies (**Figure 6A**). To test protein secretion and formation of the PvDBP-containing vaccines, vaccine-encoding plasmids were transiently transfected into HEK293E cells, and vaccine proteins in supernatant detected with a combination of C_H_3- and PvDBP-specific mAb, suggesting the fusion protein to be correctly folded and secreted as a vaccine protein (**Figure 6B**).

To assess the magnitude of the PvDBP-specific antibody responses induced by these vaccines, mice were vaccinated i.m. with a single injection of 5 µg DNA followed by EP. Antigen-specific responses were monitored for 42 days and the IgG, IgG1 and IgG2a levels were increased throughout this period (**Figure 6C**). MHCII-targeted-PvDBP DNA vaccines induced rapid anti-PvDBP-specific IgG responses that were increased above the levels induced by the non-targeted control vaccine scFv^αNIP^-PvDBP or PvDBP alone by 8 days post-vaccination (**Figure 6D**). Both dimeric vaccines (scFv^αMHCII^-PvDBP and scFv^αNIP^-PvDBP) induced rapid and increased PvDBP-specific IgG1 and IG2a responses compared to the PvDBP DNA vaccine alone on 8 day post-vaccination. These early robust responses after only one vaccination and with 10-fold less DNA compared to the PfRH5ΔNL-containing vaccines, confirms PvDBP is an immunogenic *P. vivax* antigen. In addition, these results demonstrate the compatibility of a well-described malarial blood-stage antigen from *P. vivax* to the APC-targeted DNA vaccine format.

## Discussion

Here we report a novel vaccination strategy against the merozoite form of malaria. The APC-targeting DNA vaccine, named Vaccibody, is a dimeric molecule where the antigen is genetically fused to a targeting unit. These APC-targeted vaccines, delivered as DNA, enhance antibody levels above what is detected following delivery of non-targeted versions (20, 21, 24–26, 37). We demonstrated that the leading antigen for next-generation vaccines against blood-stage *P. falciparum* malaria, PfRH5ΔNL, is conserved in this format and that APC-targeted vaccination elicits robust immune responses towards PfRH5 in mice. PfRH5 forms an invasion complex with PfCyRPA and PfRipr, and is essential to parasite invasion (7, 38). In contrast to the once-leading blood-stage vaccine antigen apical membrane antigen 1 (AMA1), which only conferred strain-specific partial efficacy due to its polymorphic nature, PfRH5 has globally limited sequence diversity (7, 39), and is shown to be sensitive for specific antibodies (11). The responses induced by the APC-targeted vaccine competed with mAb clones derived from healthy UK adult volunteers vaccinated with PfRH5, suggesting the antibody responses induced in mice to bind similar epitopes on the PfRH5 antigen. Incorporation of an additional malaria antigen, PvDBP, into the Vaccibody construct, further demonstrated that this format is also applicable to another malaria blood-stage vaccine candidate originating from the *P. vivax* parasite species.

During red blood cell invasion, PfRH5 associates with the host receptor Basigin which binds at the tip of PfRH5ΔNL at a site most distant from the C-terminus of the antigen. The C-terminal end concludes just following an α-helix, whereas the N-terminal part of PfRH5ΔNL consists of a short, two-stranded β-sheet followed by a single, short helix located at the center of the PfRH5 kite-shaped structure (10). The central location of the N-terminus in PfRH5ΔNL could potentially obscure folding and prevent access to important B cell epitopes when the antigen is connected to the dimerization unit. However, results showed that the folding of PfRH5ΔNL protein was retained within the Vaccibody format, and the immunogen contained the epitopes specified by various mouse mAbs previously characterized for PfRH5 binding and parasite growth-inhibitory activity (31). The importance of N-terminal fusion of PfRH5ΔNL within the Vaccibody was emphasized when PfRH5ΔNL was attached *via* its C-terminus to the Vaccibody molecule in the reversed Vaccibody vaccine. This reversed Vaccibody was not recognized by the same PfRH5-specific mAbs. The C-terminal fusion onto PfRH5 might have prevented correct folding of PfRH5, or the epitopes recognized by the mAbs may not have been accessible if covered by the dimerization and/or the targeting unit of the Vaccibody.

Given that antibodies only have a very short window of opportunity to bind PfRH5, high antibody concentrations are desirable to control merozoite invasion *in vivo* (13). Previous experiments in mice have demonstrated that MHCII targeting of antigen enhances Ab responses (20, 25). Additionally, MHCII targeting by vaccines is especially potent for induction of antibodies when compared to other targets on APCs (24). Intramuscular vaccination with scFv^αMHCII^-PfRH5ΔNL in the present study induced significantly higher PfRH5FL-specific IgG1 levels as compared to non-targeted control vaccines. The targeting effect observed for PfRH5FL-specific IgG1 following scFv^αMHCII^-PfRH5ΔNL vaccination was retained after repeated DNA vaccinations and even after an additional heterologous boost with the AdHu5-PfRH5FL viral vector. This is consistent with previous findings which have demonstrated that antigens targeted to MHCII molecules increase responses for most IgG subclasses, but where IgG1 is clearly dominant (25). One major advantage of this targeted response is the dose-sparing effect. A given response may be obtained by injection of a lower dose of a targeted DNA vaccine compared to non-targeted vaccines. Further, the increased responses upon several boosts with Vaccibody DNA in the present study, confirms the anti-vector responses to be low for DNA vaccines. This is an important advantage because it allows the increase of antibody levels without introducing a different vaccine booster formulation or adjuvant. However, several immunizations with DNA still preserves the opportunity to raise antibody levels with a heterologous boost.

Passive transfer of neutralizing growth-inhibitory anti-PfRH5 antibodies, independent of Fc-mediated antibody effector functions, achieved protection in non-human primates (*Aotus nancymaae*) (40). Numerous other immune effector mechanisms have also been proposed to contribute to blood-stage protection, e.g. complement activation or antibody Fc-mediated interaction with immune cells, however these have not yet been demonstrated as causative of protection in similar *in vivo* passive transfer studies. Nevertheless, the receptor targeting achieved in APC-targeted vaccines can skew the response into either a Th1 or Th2 direction *via* activation of specific subtypes of dendritic cells (24, 41). This could therefore be explored in a future approach to study antibody effector functions for protection in an *in vivo* challenge model.

The immunogenicity of PfRH5 in the context of DNA vaccination appeared rather low, as reflected by the necessity of three vaccinations to obtain robust responses versus high titers following a single vaccination with PvDBP-containing targeted vaccines. Notably PfRH5 has been reported to express relatively poorly in mammalian cell expression systems, and this observation may therefore reflect relative expression levels *in situ* post-DNA vaccination. Inclusion of PfRH5 into the MHCII-targeted DNA vaccine format may therefore have an advantageous effect on PfRH5 immunogenicity. The low immunogenicity of PfRH5 and the need for more boosts, might also explain why the targeting effect was less pronounced for IgG2a and total IgG. However, we also found a tendency of bivalency to improve responses. Indeed, the non-targeted scFv^αNIP^-PfRH5ΔNL vaccine was also better than antigen alone for total IgG (Figure 2B). The bivalency of the APC-targeted vaccines has previously been shown to be an advantage (21), increasing the amount of antigen with two antigens in one vaccine molecule. The scFv^αNIP^-PfRH5 vaccine protein will be similarly taken up by APC as the PfRH5 antigen alone. This non-specific uptake by APC is not as efficient as the targeted scFv^αMHCII^-PfRH5 vaccine, but can induce responses against the included PfRH5 antigen. Targeting of vaccine proteins to APC also results in a more efficient stimulation of CD4^+^ Th cells *in vivo* and *in vitro*, probably due to increased peptide loading of MHCII molecules (20, 25, 28). Consistent with this, in the present study, a single DNA vaccination with MHCII-targeted vaccine elicited higher PfRH5-restimulated IFN-γ responses then both non-targeted vaccines and antigen alone in dLNs and spleens as early as day 5 post-vaccination. Secretion of cytokines by the T helper subtypes affects the humoral immune responses mounted by B cells (42, 43). The characteristic Th1-cytokine IFN-γ promotes induction of IgG2a class switching, whereas the Th2-associated IL-4 drives class switching toward IgG1 in mice (44, 45). It has been reported that targeting of antigen to MHCII molecules induces a Th2-dominant response characterized by IL-4 secreting CD4^+^ T cells, although IFN-γ producing T cells are also observed (25). Increased levels of IL-4 in response to our MHCII-targeted vaccine, as compared to antigen alone, may therefore explain the improved induction of IgG1 by this vaccine format.

The *in vitro* GIA assay has been shown to correlate with *in vivo* vaccine efficacy in non-human primate studies of anti-merozoite vaccines (46–48). We showed that the APC-targeted vaccine-induced PfRH5-specific IgG was highly efficient against the 3D7 clone *P. falciparum* parasite *in vitro*. In addition, PfRH5-specific serum antibodies generated by APC-targeted vaccination are efficiently inhibited by human mAbs against PfRH5. These human mAbs bind both neutralizing and potentiating epitopes on PfRH5 that are conserved across all tested strains of *P. falciparum* (11). Consequently, our targeted vaccine elicits antibodies relevant for human antibody responses against PfRH5. In addition, DNA plasmids has a relatively high resistance against degradation, thus the DNA vaccine format is relatively independent of a cold chain for distribution. The cost-efficient production, as well as easiness to produce in large amounts (17), makes DNA vaccines an attractive vaccine platform. We also showed that this platform induced efficient antibodies for another malaria blood-stage vaccine candidate originating from *P. vivax* parasites, PvDBP, suggesting the vaccine format presented in this study represents a novel and attractive alternative for the development of vaccines to control blood-stage malaria.

## Data Availability Statement

Data not included in the article will be made available upon request to the corresponding author. There were no datasets generated in the current study. Requests to access the datasets should be directed to RB, ranveig.braathen@medisin.uio.no.

## Ethics Statement

The animal study was reviewed and approved by the Norwegian food safety authority (Mattilsynet, FOTS ID 19555).

## Author Contributions

LB, GV, SD, BB, and RB conceived the study, designed the experiments, and analyzed the data. LB, GV, AG, GL, DQ, DP, HS, and RB performed the experiments, carried out the data collection and data analysis. HS and DP contributed to reagents and materials. LB, SD, BB, and RB wrote the article and revised the manuscript. All authors contributed to the article and approved the submitted version.

## Funding

This work was supported by grants from Research Council of Norway, Globvac grant 234431 (to BB), and Innovation grant 2018 from University of Oslo (to RB). SD is also a Jenner Investigator and a Wellcome Trust Senior Fellow [106917/Z/15/Z]. The funders had no role in study design, data collection and analysis, decision to publish, or preparation of the manuscript.

## Conflict of Interest

SD is a named inventor on patent applications relating to RH5 and/or other malaria vaccines and immunization regimens. BB and RB are inventors on patent applications filed on the vaccine molecules by the Technology Transfer Offices of the University of Oslo and Oslo University Hospital, according to institutional rules. BB is head of the scientific panel of Vaccibody AS. The other authors have no financial conflicts of interest.

The remaining authors declare that the research was conducted in the absence of any commercial or financial relationships that could be construed as a potential conflict of interest.

## Publisher’s Note

All claims expressed in this article are solely those of the authors and do not necessarily represent those of their affiliated organizations, or those of the publisher, the editors and the reviewers. Any product that may be evaluated in this article, or claim that may be made by its manufacturer, is not guaranteed or endorsed by the publisher.

## Abbreviations

APCs, antigen presenting cells; AMA1, apical membrane antigen 1; EP, electroporation; HEK, human embryonic kidney; i.m., intramuscular; i.p., intraperitoneal; MHCII, major histocompatibility complex class II; n.d., not detected; PfRH5, *Plasmodium falciparum* reticulocyte-binding protein homolog 5; PvDBP, *P. vivax* Duffy-binding protein*;* scFv, single chain fragment variable.
