# Supplementary material for: APC-Targeted DNA Vaccination Against Reticulocyte-Binding Protein Homolog 5 Induces Plasmodium falciparum-Specific Neutralizing Antibodies and T Cell Responses
Source: Front Immunol. 2021 Oct 18;12:720550. doi: 10.3389/fimmu.2021.720550 (PMC8558525; doi:10.3389/fimmu.2021.720550)
Supplement: Supplementary file 1 [file DataSheet_1.pdf]

Supplementary Figures

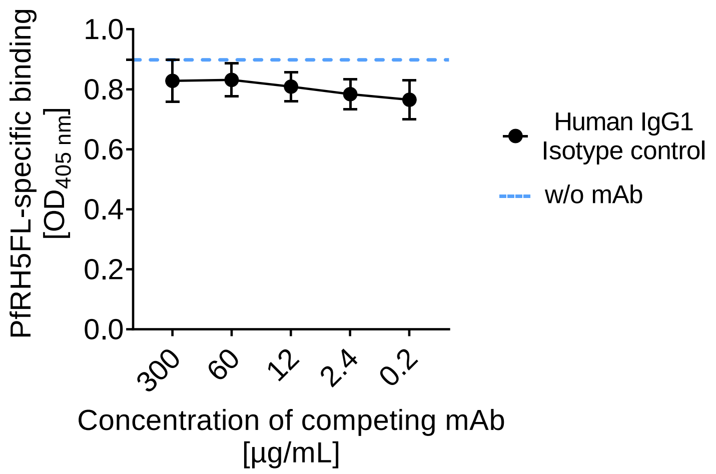

Supplementary Figure 1. Isotype control do not prevent binding of PfRH5-specific sera to PfRH5 protein. BALB/c mice (n = 6) were vaccinated three times (day 0, 21 and 42) with 50 µg DNA i.m./EP with scFv$^{\alpha MHCII}$-PfRH5ΔNL and sera harvested at day 62. Pooled sera were incubated with a titration (50, 10, 2, 0.4 and 0.08 µg/ml) of a human IgG isotype control antibody or without (w/o) mAb (stipled line) before binding to PfRH5FL protein were analyzed in ELISA. Samples were performed as technical triplicates (mean ± SD).

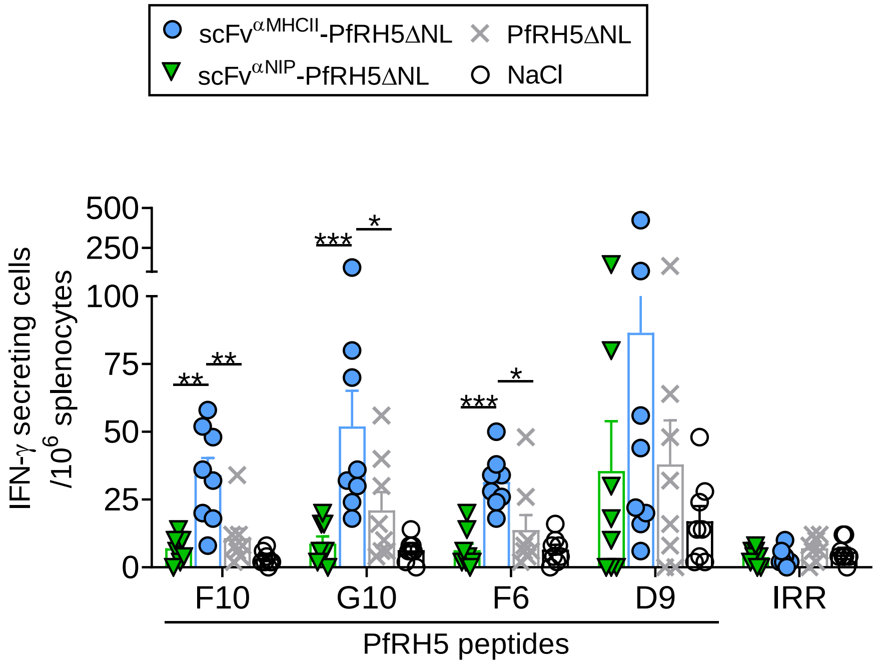

Supplementary Figure 2. PfRH5ΔNL-containing targeted DNA vaccines induced rapid IFN-γ T cell responses against PfRH5 peptides. BALB/c mice were immunized i.m./EP with 50 µg of indicated DNA plasmids or NaCl. Spleens were harvested at day 5 post vaccination and IFN-γ producing splenocytes were measured by ELISPOT after stimulation with the indicated PfRH5 peptides or irrelevant control (IRR, IYSTVASSL from hemagglutinin). n = 8 mice/group (mean ± SEM). *p<0.05, **p<0.01, two-tailed Mann-Whitney test.
